# Upper extremity physical performance tests in female overhead athletes: a test–retest reliability study

**DOI:** 10.1186/s13018-023-03974-4

**Published:** 2023-07-10

**Authors:** Sara Kardor, Zahra Gorji, Nastaran Ghotbi, Behrouz Attarbashi-Moghadam, Azadeh Shadmehr, Mona Gorji

**Affiliations:** 1 Department of Physiotherapy, Faculty of Rehabilitation, Tehran University of Medical Sciences, Tehran, Iran; 2 Skin Research Center, Shahid Beheshti University of Medical Sciences, Tehran, Iran

**Keywords:** Reliability, Athletes, Upper extremity, Stability test, Power test, Bland–Altman plot

## Abstract

**Objective:**

Despite the studies that have investigated the reliability of Upper Extremity Functional Tests**(**UEFTs), the reliability of Closed Kinetic Chain Upper Extremity Stability(CKCUES), Seated Medicine Ball Throw(SMBT), push-up(PU) and Unilateral Seated Shot Put(USSP) tests in overhead athletes has yet to be assessed. The objective of this study was to determine both the relative and absolute test–retest reliability of the four UEFTs in female overhead athletes.

**Methods:**

Twenty-nine female overhead athletes (age: 26.6 ± 5.29 years) underwent the four UEFTs twice within a three- day interval. The upper limb stability was assessed through PU and CKCUES tests, while the power was assessed though SMBT and USSP tests. The Intraclass Correlation of Coefficient (ICC) was applied to assess the relative reliability. Absolute reliability was determined by calculating the Standard Error of Measurement (SEM) and the Minimal Detectable Change (MDC). Furthermore, Bland–Altman plots were used to detect the agreements between the two measurements.

**Results:**

The relative reliability of PU, CKCUES, SMBT, and non-dominant arm USSP tests was excellent (ICC = 0.83, 0.80, 0.91, and 0.83, respectively). SEM was within a range of 1.69 to 1.72 for stability tests and a range of 13.61 to 52.12 for power (based on a 95% confidence interval). The MDC was 4.68 for PU and 4.75 for CKCUES test. At least four repetitions are needed to be considered a real improvement on PU and CKCUES tests. This value was 144.04, in SMBT and 59.03, 37.62 cm (dominant and non-dominant arm, respectively) in USSP tests, which represents the minimum change that must occur to be considered an athlete’s progression.

**Conclusion:**

This study revealed that both the upper limb stability and power tests have acceptable relative and absolute intra-rater reliability in female overhead athletes. These can be considered as reliable tools in research and clinical settings.

## Introduction

The shoulder complex is a collective system of four interconnected joint articulations, each of which plays an important role in the shoulder range of motion (ROM). As the joints are interconnected, no single muscle can act alone in the shoulder complex and many related muscles must be involved to accomplish the movement of concern.

Overhead athletes are exposed to shoulder injuries due to the forceful and repetitive nature of their movements [1, 2], especially in throwing sports like baseball, volleyball, and badminton. The throwing shoulder must be sufficiently loose to allow a 180-degree flexion or abduction and yet stable enough to prevent injuries [1, 3]. If athletes returns to sport without a proper rehabilitation program concerning their functional stability and mobility, the risk of re-injury will be high. So, an accurate physical examination is necessary to make a delicate decision on the recovery of athletes following different shoulder injuries [4, 5]. Physical examinations should detect functional and biomechanical impairments in professional or daily life activities. Assessing ROM and muscle strength are standard components of physical examinations, yet they may not be sufficient in providing comprehensive information. To overcome this drawback, upper limb functional tests have been developed. These tests are many, some demonstrate stability and some mobility and power features of the shoulder. Consequently, they can provide quantitative evidence for the success and efficacy of rehabilitation programs [1, 2]. Among the tests that validated for measuring the functional stability of the upper extremity, Closed Kinetic Chain Upper Extremity Stability (CKCUES) and Push-up (PU) tests are more common since they are easily understood and administered, cost-effective, and are acceptable alternatives to the bench press repetitions-to-failure test [4, 6].

On the other hand, upper extremity power, as one of the essential properties of overhead athletes, can be assessed through Seated Medicine Ball Throw (SMBT) and Unilateral Seated Shot Put (USSP) tests [7]. Due to their feasibility, these tests are administered frequently to quantify upper body explosiveness in the practical setting.

However, due to the specific nature of all mentioned tests, clinicians and researchers select each test based on their specific assessment purposes. In other words, SMBT and USSP tests are used for the assessment of the upper extremity power; whereas CKCUES and PU tests are more suitable for neuromuscular control and stability evaluation of the upper limb. Therefore, it seems that an isolated performance test cannot accurately assess shoulder function in overhead athletes to decide on time to return to sport [8].

If an athlete returns to play without a proper rehabilitation program regarding their functional stability and mobility, the risk of re-injury will be high. Return to sport needs an accurate, precise and multi-dimensional assessment of the shoulder function. Although studies have shown that there is no consensus on criteria for returning to sport after shoulder injuries, in some studies, ROM, muscle strength, and time after injury are used as criteria. However due to the complexity of the shoulder joint, these criteria are not sufficient, and using the four functional tests can provide good information about shoulder function and athletes’ readiness to return to sport [9].

Isokinetic dynamometers are standard measurement equipment that objectively assess muscle strength as another important criterion. However, using them in non-laboratory settings is not feasible, so clinicians need an alternative assessment test such as SMBT [8].

Since Upper Extremity Functional Tests (UEFTs) are widely applied in different populations; many researchers have assessed their reliability. Goldbeck and Davies conducted the relative reliability of the CKCUES test on 24 male college students and revealed the test–retest intraclass correlation coefficient (ICC) to be 0.92 [4]. In studies conducted on men and women with and without shoulder pathologies, the relative reliability of CKCUES test was found excellent [10, 11]. Moderate to excellent reliability of CKCUES test was observed in adult and adolescent populations [12, 13]. Negrete et al. [5] found high reliability of modified pull-up, PU, and single arm shot put tests on healthy recreationally active adults. Davis et al. and Harris et al. reported high reliability of SMBT test in older adults and kindergarten children [14, 15].

Unlike the mentioned studies, the reliability of CKCUES, SMBT, PU, and USSP tests in overhead athletes, has not been assessed yet. In addition, most studies have only measured the relative reliability through ICC which cannot determine the measurement errors due to repeated assessments. The absolute reliability of Standard Error of Measurement (SEM) and Minimal Detectable Change (MDC), is very important especially when the tests are run as a measure of performance improvement or clinically significant [10].

Therefore, the purpose of the present study was to determine both the relative and absolute test–retest reliability of four UEFTs in overhead athletes. Furthermore, the Bland–Altman method was adopted to reveal the agreement between the measurements, which was applied only in a few studies. The findings of this study would assist clinicians to choose appropriate tests and interpret the clinical data of overhead athletes.

## Methods

*Design* The present research was conducted with a cross-sectional and methodological design. Written informed consent was signed by all participants and the study was approved by the Ethics Committee of the School of Rehabilitation of Tehran University of Medical Sciences. The following equation was applied to calculate the sample size:

$$C_{r} = {\raise0.7ex\hbox{$1$} \!\mathord{\left/ {\vphantom {1 2}}\right.\kern-0pt} \!\lower0.7ex\hbox{$2$}}\;Ln\;\frac{1 + r}{{1 - r}},\;n = \left( {\frac{{Z_{{\left( {1 - {\raise0.7ex\hbox{$\alpha $} \!\mathord{\left/ {\vphantom {\alpha 2}}\right.\kern-0pt} \!\lower0.7ex\hbox{$2$}}} \right)}} + Z_{{\left( {1 - \beta } \right)}} }}{{C_{r} }}} \right)^{2} + 3$$

*Participants* Twenty-nine female overhead recreational athletes (10 volleyball, 9 basketball, and 10 badminton players), within a range of 18–35 years volunteered to participate in the study (age 26.6 ± 5.29 years, height: 166.31 ± 5.6 cm, weight: 61.72 ± 10.54 kg) and were tested twice with a three-day interval period to decrease the likelihood of training effects.

The inclusion criteria were (1) female athletes aged 18 to 35, (2) active in overhead sports without any history of upper limb trauma or injury that had limited activity for more than two consecutive days in the past six months, and (3) without any systemic and neuromuscular disease. The exclusion criteria were a history of upper extremities or spinal surgery, and the use of hypnotic and sedative medications. The subjects were also excluded if they reported a new injury within the first two days of the test period, and pain or fatigue following the first trial [16, 17].

*Procedures* First, tests were completed in a random order, and then the athlete was instructed to perform the tests. Before each test, a five-minute routine warm-up plus 3 min of anterior shoulder stretch, horizontal adduction for posterior shoulder stretch, and trunk side-bending overhead reach were performed to stretch the trunk and inferior shoulder girdle [18–20].

### Push-up test

**Fig. 1:**
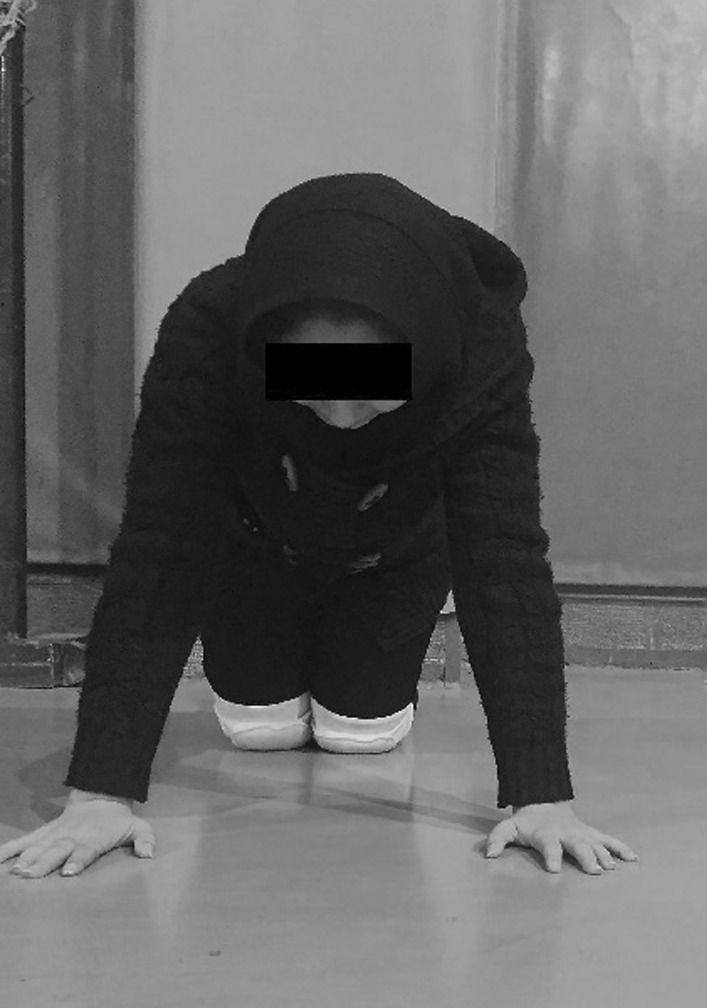
Starting position for push-up

The athletes assumed the modified PU position with their hands placed under their shoulders, fingers pointed forward, and elbows pointed backward (Fig. 1). The participants first held the position with a PU on full arm extension so their body weight would rest on their hands and toes. Then the athletes lowered themselves until all their body from the chest to the thighs made contact with the floor. Completing this process was counted as one successful PU test. The athletes completed as many repetitions as possible during 3 sets and rested 45 s between sets. One examiner controlled the stopwatch, and the other counted the touch count [5]. The PU score was the number of correct Pus [4, 5].

### Closed kinetic chain upper extremity stability test

**Fig. 2:**
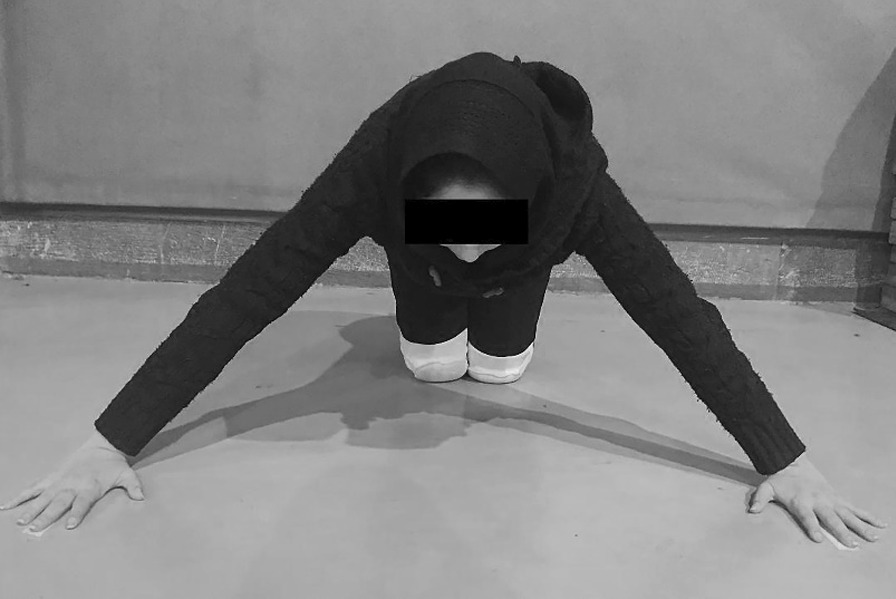
Starting position for closed kinetic chain upper extremity stability test (CKCUEST)

During this test, the athletes performed the modified PU position (with knee support), and both hands placed on two adhesive tape markers affixed to the ground at a distance of 91.4 cm (Fig. 2). The athlete remained in the modified PU position with one hand on each piece of tape. Then, for 15 s, the athletes alternatively touched the opposite hand. The hand touch count is the score for this test. The athletes completed as many repetitions as possible during 3 sets and rested 45 s between each set. One examiner controlled the stopwatch, and the other the touch counts [4, 21].

### Seated medicine ball throw test

**Fig. 3:**
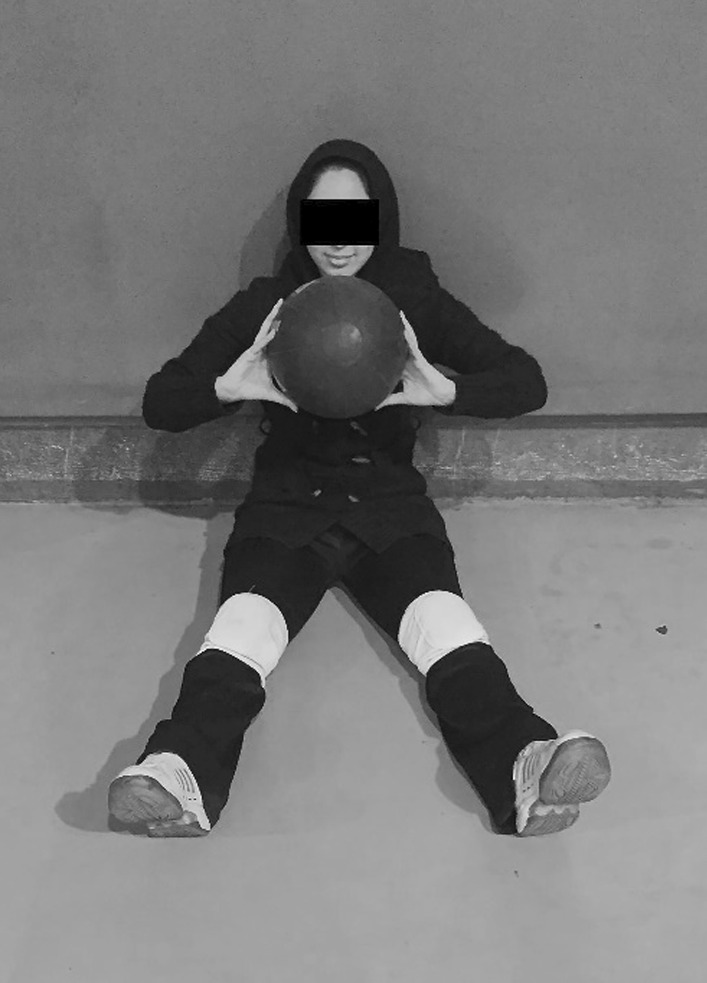
Starting position for seated medicine ball throw (SMBT) test

This test was performed in a sitting position with a 2 kg medicine ball and a measuring tape. Athletes sat on the floor with their backs against a wall for support and their legs stretched out. Participants began by holding a 2 kg medicine ball with both hands and resting it on their chest (Fig. 3). They threw the ball with a chest press motion for distance. This distance was measured and recorded with the same measuring tape. To minimize the error, each participant was allowed three attempts with an interval of 1 min between each throw. One examiner measured the throw distance, and the other checked the athlete’s position [15, 22, 23].

### Unilateral single-arm shot-put test

**Fig. 4:**
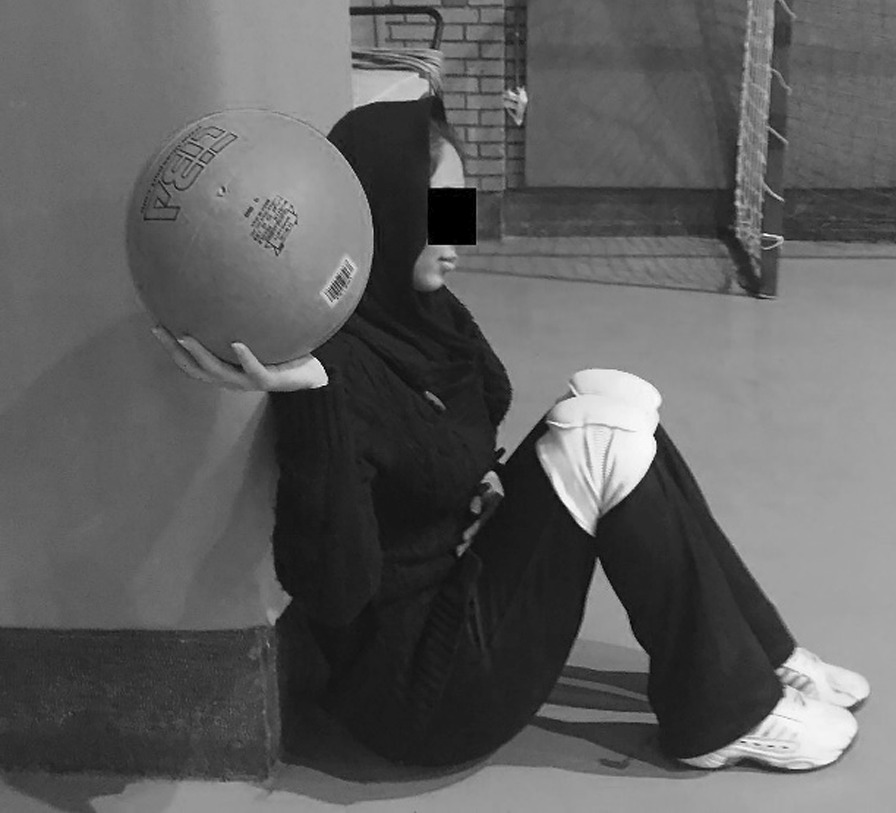
Starting position for unilateral seated shot-put (USSP)Test

This test was performed using a 3 kg medicine ball [5, 24]. Athletes sat on the floor with their backs against the wall, knees bent at 90 degrees, and feet resting on the floor. The athletes were placed next to a doorway to allow unrestricted arm movement on the test side. Participants were instructed to hold the medicine ball at shoulder level and push it (not to throw) as far as possible (Fig. 4). The participant’s head and back were in contact with the wall and their non-engaged arm was on their lap. After 1 min, the test was repeated for the other side [24]. One examiner measured the ball's distance, and the other checked the athlete’s position.

All four UEFTs were repeated three times and the mean was calculated. The subjects rested for 3 min between each main test.

### Statistical analysis

SPSS version 23.0(SPSS Inc., Chicago, IL, USA) was used for data analysis and significance was set at *p* < 0.05. The normality of distribution was assessed by the Kolmogorov–Smirnov test. The relative reliability was calculated using ICC_(2, 1)_. ICC values above 0.75 were considered excellent reliability. Values between 0.40 and 0.74 represented moderate while below 0.40 showed poor reliability[25].

Absolute reliability, expressed as SEM, was evaluated to determine the precision of each score. MDC was also calculated to estimate the threshold for measurement error. The MDC is the minimum change in score that an individual must experience to ensure that the change in score is not simply due to measurement error.

The equations of these tests were:

$${\text{SEM}} = {\text{ SD}} \times \sqrt {\left( {{1} - {\text{ICC}}} \right)}$$

$${\text{MDC}} = {\text{SEM }} \times { 1}.{96} \times \sqrt 2$$

Then MDC percentage was calculated by dividing MDC into the maximal score of the measurement. MDC percentages less than 30 and 10 are considered acceptable and excellent, respectively. For SEM, the values equal to 0 are considered perfectly reliable while the values equal to SD are interpreted as completely unreliable [26]. The coefficient of variation (CV) was calculated as the SD to the mean ratio (CV = SD/Mean × 100) to show the extent of variability.

A paired *t*-test was also used to show the differences in the mean scores between the two test sessions. The Bland and Altman chart was plotted for each test to provide a visual interpretation of the agreement between the two assessment sessions.

## Results

The demographic information of the athletes are presented in Table 1. No significant difference between test sessions for either the stability or power tests (*P* > 0.05) was observed in the paired *t*-test (Table 2), revealing that there is no systematic bias.

**Table 1:** Demographic characteristics of study populations (*n* = 29)

Variable	Mean	SD	Minimal	Maximal
Age	26.6	5.29	18	35
Weight(kg)	61.72	10.54	46	98
Height(m)	166.31	5.6	156	177

SD: Standard Deviation

**Table 2:** Paired *t*-test comparing test–retest scores among upper extremity functional tests

Test	Mean ± SD First session	Mean ± SD Second session	Mean difference	*p* value
PU	12.74 ± 4.14	13.73 ± 3.76	− 0.99	0.08
CKCUES	15.77 ± 3.91	17.06 ± 4.8	− 1.29	0.06
SMBT	375.31 ± 55.45	366.41 ± 55.6	8.9	0.14
USSP(dominant arm)	256.28 ± 36.21	262.24 ± 45.01	− 5.97	0.44
USSP (non-dominant arm)	235.59 ± 33.21	241.83 ± 35.05	− 6.24	0.2

PU: Push-up, SMBT: Seated medicine ball throw, CKCUES: Closed kinetic chain upper extremity stability, USSP: Unilateral seated shot put

### Relative reliability

ICC values of all tests were within a range of 0.65 to 0.91, suggesting moderate to excellent reliability. The PU, CKCUES, SBMT, and USSP (non-dominant hand) tests had excellent reliability, and the USSP test in the dominant hand showed moderate reliability (Table 3).

**Table 3:** Test–retest reliability indices of the functional tests

Test	ICC (95% CI)	SEM	MDC (MDC %)	CV(%)
PU	0.83 (0.65–0.92)	1.69	4.68 (21.27)	5
CKCUES	0.8 (0.58–0.9)	1.72	4.75 (20.36)	5
SMBT(cm)	0.91 (0.81–0.95)	52.12	144.04 (29.88)	1
USSP (dominant arm)	0.65 (0.26–0.72)	21.36	59.03 (18.38)	1
USSP (non-dominant arm)	0.83 (0.65–0.92)	13.61	37.62 (12.54)	1

PU: Push-up, SMBT: Seated medicine ball throw, CKCUES: Closed kinetic chain upper extremity stability, USSP: Unilateral seated shot put

### Absolute reliability

**Fig. 5:**
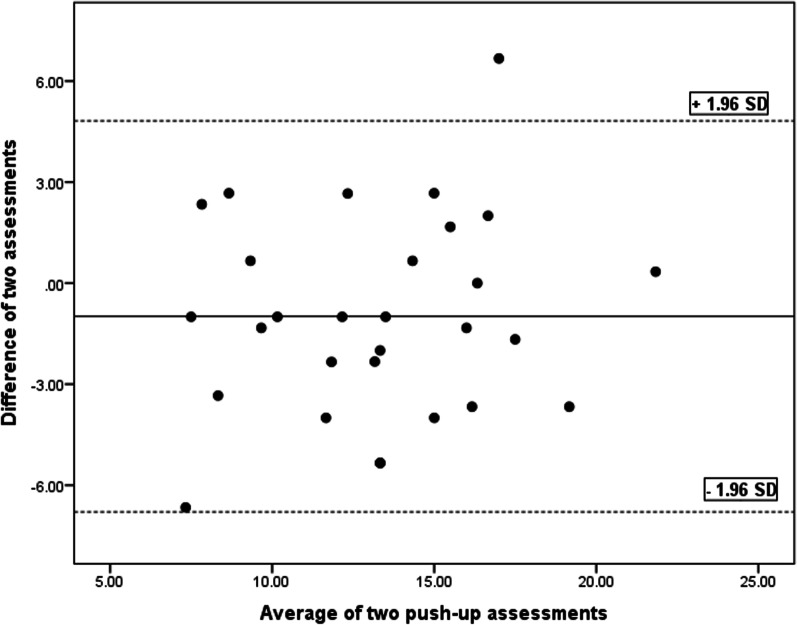
Bland–Altman plot for push-up test

**Fig. 6:**
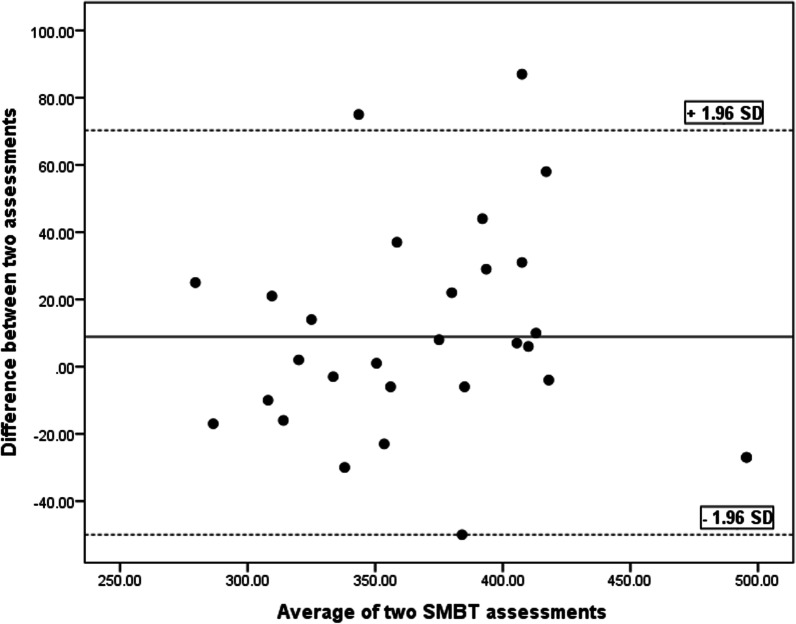
Bland–Altman plot for seated medicine ball throw (SMBT) test

**Fig. 7:**
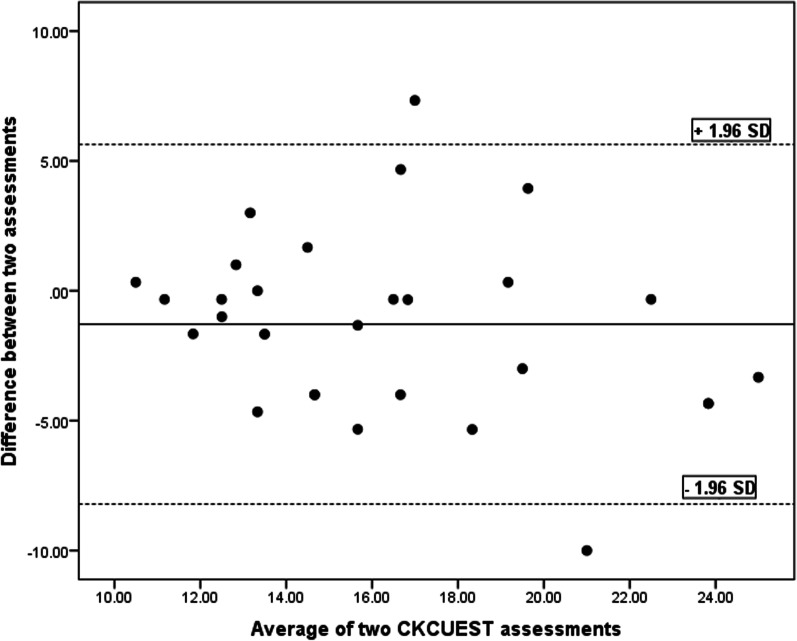
Bland–Altman plot for closed kinetic chain upper extremity stability test (CKCUEST)

**Fig. 8:**
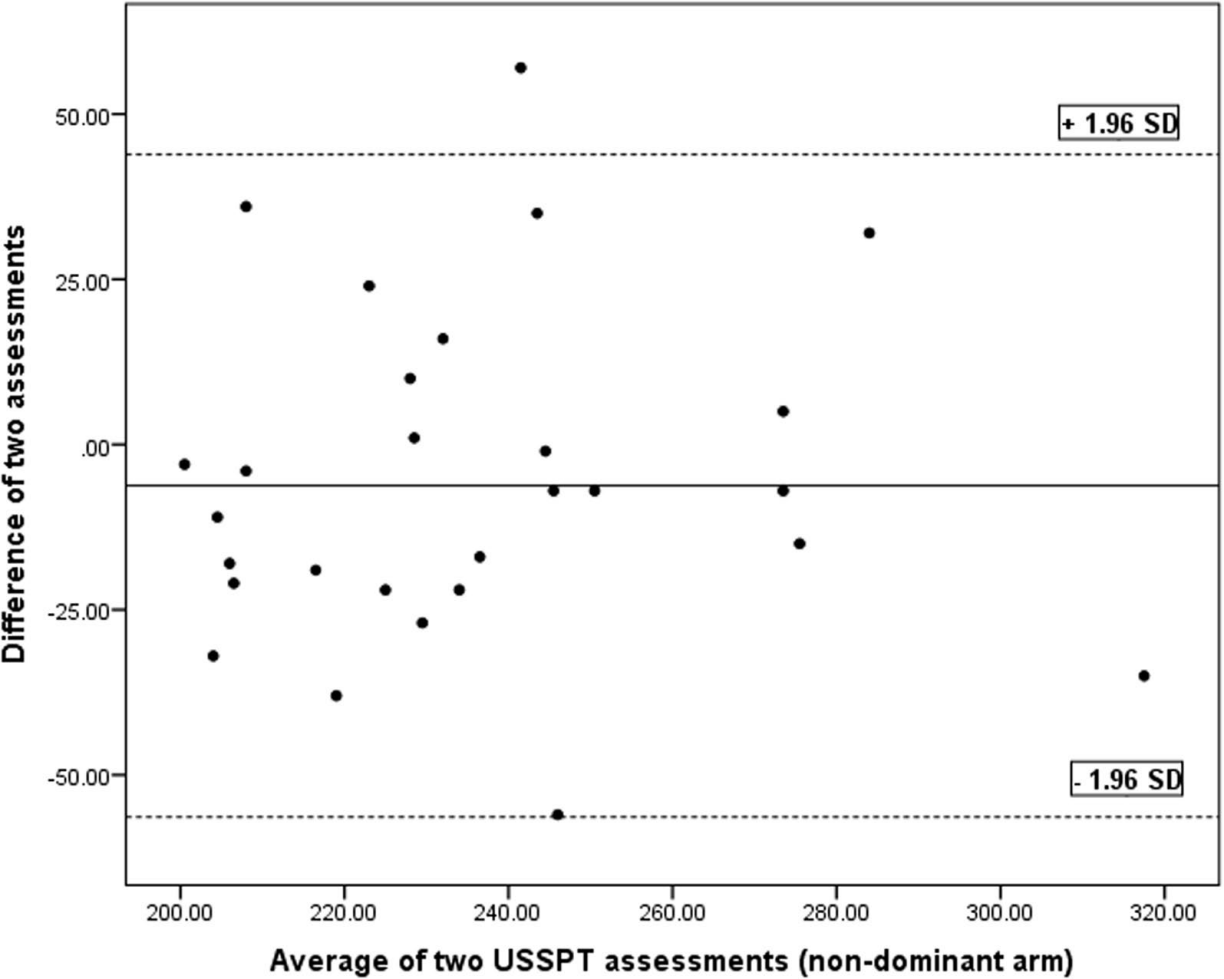
Bland–Altman Plot for unilateral seated shot put (non-dominant arm) test

**Fig. 9:**
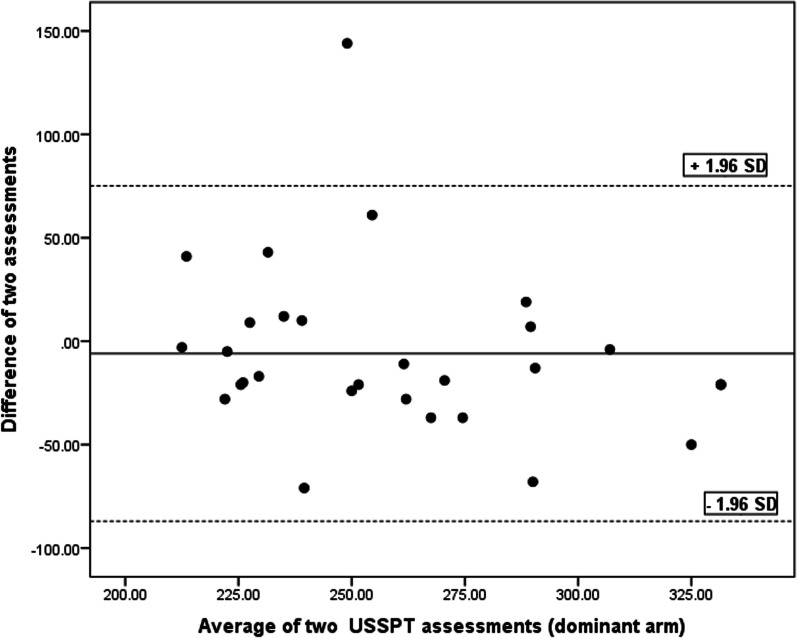
Bland–Altman Plot for unilateral seated shot put (dominant arm) test

SEM, MDC and CV values are tabulated in Table 3. The Bland and Altman charts are demonstrated for the four functional tests in Table 4 and Figs. 5, 6, 7, 8 and 9.

**Table 4:** Bland and Altman analysis of the tests

Test	Mean diff.(d)	SE of d	95% CI of d	Limits of agreement
PU	− 0.98	2.97	− 2.11–0.14	− 6.79–4.81
SMBT	8.89	31.33	− 3.02–20.81	− 52.52–70.31
CKCUE	− 1.28	3.53	− 2.63–0.53	− 8.21–5.63
USSP (dominant arm)	− 5.96	41.35	− 21.69–9.76	− 87.02–75.09
USSP (non-dominant arm)	− 6.24	25.57	− 15.96–3.48	− 56.35–43.87

PU: Push-up, SMBT: Seated medicine ball throw, CKCUES: Closed kinetic chain upper extremity stability, USSP: Unilateral seated shot put

## Discussion

The objective of this study was to determine the test–retest reliability within a three-day interval for PU, CKCUES, SMBT, and USSP tests in female overhead athletes. Due to the specific property of the test–retest reliability, we assessed ICCs (95% CI), SEM, MDC, and CV. The mean scores and differences in test indices between the two measurement sessions were assessed by paired *t*-test. Bland and Altman’s plots were demonstrated. Generally, to our knowledge, the novelty of this study was (1) simultaneous assessment of reliability for two stability and two power upper limb functional tests, (2) reliability assessment in female overhead athletes, and (3) comprehensive assessment of the reliability by assessing the six parameters/methods.

The findings of this study showed excellent relative reliability for both the stability and power tests except for the dominant arm USSP test which showed moderate reliability. The findings of relative reliability for PU, CKCUES, and SMBT tests were in agreement with that of Harris, Davis, Negrete, Sciascia, and Goldbeck et al., where only one or some of the above tests were assessed [4, 5, 11, 14, 15]. In the present study, the pushing distance during USSP test increased from 256.28 to 262.24 cm in the dominant hand and from 235.9 to 241.83 cm in the non-dominant hand, making ICC moderate in the dominant hand and excellent in the non-dominant hand. These values were less those reported by Negrete et al. and Degoat et al. [20, 27], which may probably be due to the difference in throwing technique and the statistical population. In their study, recreationally active adults threw the ball while sitting on a chair, but in our study, athletes sat on the floor. The ball weight and the athletes’ gender are different in these two studies as well. The result of the present study was in agreement with Davis for SBMT test that showed medicine ball throw is highly reliable within 1 day (ICCs = 0.93) [14]. For CKCUES test, our findings were in agreement with Goldbeck that showed the ICC was 0.922 for test–retest reliability and reported CKCUES test as a reliable evaluation tool [4].

The dominant shoulder of overhead athletes is different from that of the non-athletes and the type of each overhead activity as well [28]. The ICC for SMBT test was excellent in this study and in agreement with and Beckham, Koo and Li, Gillespie, and Kenum et al.’s findings. The correlation of these results revealed that distance measurement through SBMT test is a reliable test for assessing upper limb power [29–31].

Good ICC for PU test was found to be within a range of 0.65 to 0.92 in this study, which is supported by Bohannon’s 0.87 to 0.90 range and Gillen et al.’s range of 0.65 to 0.79[32].

### Absolute reliability

The SEM value for PU and CKCUES tests was 1.69 and 1.72, respectively. The MDC values in PU and CKCUES tests were 4.68 (21.27%), and 4.75 (20.36%), respectively. These values would assure clinicians that at 95% confidence, the changes over 4 repitations were considered the true improvement. In general, the lower the SEM and MDC values, the more reliable the measurements[33].

The values obtained through SEM for *CKCUES* test were close to those of Sciascia et al. and Tucci et al. at 2 and 2–2.76 touches, respectively. As MDC, the same holds for findings at 4 and 2.82–3.91, respectively [10, 11]. In this study, the SEM and MDC values for the PU test were greater than that of Negrete et al.’s study (1 and 2 repetitions), respectively, which involved both genders [20]. The female athletes in this study took the test in a modified position (putting their knees on the floor).

The SEM value in *SMBT* in our study (52.12 cm) was greater than the one found by Harris et al. (14.8–19.1 cm) and by Beckham et al. (14 cm) [15, 29].

This could be related to the difference in age and athletic performance of the participants [15]. The difference in ball weight is another factor. The MDC value was absent in Harris et al. [15] and Beckham et al.’s [29] findings.

The SEM values in this study for dominant and non-dominant *USSP* were 21.36 and 13.61, and the MDC values were 59.03 and 37.62, respectively. Regarding SEM and MDC, there existed only 3 studies where these values in addition to the ICC were of concern. These values were different from our study, which could be related to the reasons discussed earlier in the relative reliability section. The typical error (or the coefficient of variation percent) for SMBT and USSP (dominant and non-dominant hand) was 1%, while PU and CKCUES tests had a 5% error. All the tests in this study had values lower than 10%, which is considered valid. This is in line with Degot’s study which found a 5.02% error for USSP test [27]. The mean CV for the first and second trials of SBMT test was 4.2%, a higher result than ours [29]. The CV for CKCUES test was reported in the Degot’s study to be 10.3% which is not commonly observed in other studies. This value was significantly higher than the one obtained in our study [34]. The lower values of CV in this study compared to the mentioned studies may be attributed to the population. In other words, in the present study only women participated and all were in the same sport category, i.e. overhead sports.

### Bland–Altman analysis

This analysis is a supplement for the other reliability indices (ICC, SEM, MDC, and CV). To the researchers’ knowledge, this analysis was run in a few studies to determine the reliability of the four functional tests in overhead athletes [13, 27, 29, 35, 36]. As observed in Figs. 5, 6, 7, 8 and 9, there exists one outlier for PU and both USSP tests and two outliers for CKCUES and SMBT tests. In this study, according to USSPT, the higher score (further throw) indicates less agreement. In the case of CKCUES, the lower score (fewer touches) indicates better agreement.

Future studies are needed to compare the reliability of women overhead athletes with men and to assess the reliability of these tests in other athletic disciplines like gymnastics and wrestling which use weight-bearing positions. In this study, the tests were not normalized to body dimensions (e.g. weight and height). It is suggested that future studies consider this issue.

## Conclusions

The four upper extremity functional tests have excellent reliability in overhead athletes, except for the dominant USSP test, which revealed moderate reliability. These low-cost, easy-to-run tests would help the sports clinicians in assessing the shoulder function and athletes’ sport readiness.

Based on the absolute reliability findings, the clinicians can apply the reported scores here as a reference to detect the real improvement in either a group or a single female overhead athlete(s).

### Clinical relevance

- Push-up test, Closed Kinetic Chain Upper Extremity Stability test, Seated Medicine Ball Throw test, and Unilateral Seated Shot Put test (non-dominant hand) have excellent reliability.
- Sports clinicians can apply these low-cost, easy-to-operate tests to examine the improvement of the athletes or patients with similar dysfunctions
- The clinicians can apply the scores found here as a reference in detecting the real improvement in either a group or a single female overhead athlete(s).
